# Measurement on physical parameters of raindrop energy

**DOI:** 10.1186/2193-1801-2-S1-S16

**Published:** 2013-12-11

**Authors:** Minghang Guo, Jinshi Jian, Zhun Zhao, Juying Jiao

**Affiliations:** Institute of Soil and Water Conservation, Northwest A & F University, Yangling, China; Resource and Environment College, Northwest A & F University, Yangling, 712100 China; Institute of Soil and Water Conservation, Chinese Academy of Sciences and Ministry of Water Resources, Yangling, China

**Keywords:** Rainfall erosivity factor (R), Raindrop energy (E), physical parameters of raindrop, photogrammetric measurement

## Abstract

Rainfall erosivity factor (R) is one of the most commonly used factors in soil erosion models. While rainfall energy (E) is the most elementary physical parameter to predict R. Based on comparative analysis of previous soil erosion models and rainfall erosivity factor measuring methods, integrated application of modern photogrammetric techniques, image analytic methods and automatic control theories, this paper provided a new method based on image analytic to calculate the rainfall energy and R factor, which obtains raindrop's volume and velocity by means of modern photogrammetric technique. Results show that this method can improve both efficiency and accuracy of rainfall energy calculation and other rainfall physical parameters measurement.

## Introduction

Soil erosion models research is a front field of soil erosion subject, frequently, rainfall factors are indispensable element at any model, R is one of the most frequently used factor. After comparing with various models, a conclusion was found that rainfall energy, rainfall intensity (I) and rainfall precipitation (P) are the most widely used rainfall parameters. Among these parameters, I and P can be acquired through observational datas from weather stations, while there are not yet an observing method to obtain rainfall energy, which is usually calculated through statistic models. Fortunately, with the development of modern high technology, especially the development of modern photogrammetric technique, image analytic method and automatic control theory, physical parameters of rainfall could be measured directly, and it is of great valuable to calculate the rainfall based on these physical parameters, which make it possible to obtain rainfall energy by measuring instead of statistic models. No doubt, it would be of great significance to soil erosion subject's experimental techniques innovation.

## Presentation of question

### Calculation of rainfall energy

The concept of R was firstly put forward by American scholars Wischmeier W. H and Smith D. D et al [1], After comprehensive analysing the relationship among soil loss data and many rain physical parameters such as P, E, maximum time intensity (IN), antecedent precipitation (Pa) and other compound factors, a formula was established by they to calculate R value:1

Where E is the total energy of a rainfall (MJ/hm^2^), and I_30_ the maximum 30-min intensity (mm/h).

As the USLE has widely been used for predicting soil loss in countries all over the world, the formula (1) is generally recognized as one of the most classic calculation methods to predict R [12].

E is determined by quality and velocity of raindrop, as a result, a rain drop's energy can be calculated if quantity and velocity are known, an individual rainfall's energy is the sum of all drops. Energy of an individual rainstorm is the function of the total rainstorm quality and the various rain intensity [13], the following equation was used to calculate the energy of an individual rainstorm:2

Where e is the energy of one unit rainfall (J), i is rainfall intensity of differential time (mm/min), t is differential time and T is the total time of one rain.

However, as the physical shape and velocity of one rain drop is hardly to measure, so far, energy of rainfall is still acquired by the relationship between energy and statistic rain intensity. In most cases, formula (2) can be replaced by this following discontinuous form:3

Where E is the energy of a certain period rainfall (MJ/hm^2^), e the energy of per unit rainfall (MJ/hm^2^ mm) and P the rainfall precipitation of one certain period. In the Wischmeier W. H's formula, e can be determined by the formula as follows:

Where e is the energy of per unit rainfall (J/m^2^ mm) and I the intensity of per unit rainfall (mm/h).

Through analyzing nature raindrops' characters, the calculation formula of Northwest, Northeast and Southern of China were established respectively by Zhongshan Jiang [14], Suyuan Liu [15], Fujian Zhou [16] to calculate the rainfall energy, which is express as, e=a+blogi or e=ai^b^, where e is the energy of per unit rainfall(J/m^2^ mm), i is the rainfall intensity, a and b is calculating coefficients.

As a result, how to determine the rainfall energy (e) and intensity (I) of one unit period is the key to measure and calculate the energy. Between these two parameters, I is a common rainfall character value which can be obtained by measuring rainfall precipitation and rainfall duration, while the measurement and calculation of energy is relatively difficult.

### The traditional ways to measure raindrop's quantity and down velocity

One way to measure rain drop's quantity is called Stain method (or filter paper method) [17]. The precise process are as follows: firstly, a piece of filter was spread with water-solubled dyestuff, which doesn't show color when dry but a permanent rough circular stain will emerge when it was wetted by raindrops. Then, every stain's diameter was measured, raindrop's diameter can be determined according to a relationship between diameter of drops' and stain's. At last, their quantity can be calculated according to their volume if they are regard as sphere. The velocity of raindrops is distinguished by two situations [18], when a drop's diameter < 1.9 mm, velocity is calculated according to amendatory Sha Yuqing formula, when a drop's diameter ≥ 1.9 mm, calculated by amendatory Newton formula. An individual drop's energy can be computed based on moving object's energy formula when a drop's quantity and velocity are known, the unit area's rainfall energy of an individual rainfall is the sum of every single raindrop's energy.

From these above current classic rainfall energy calculation, it can be concluded that the R factor of an individual rain is determined by energy and intensity, intensity can be computed through measured data by self-restrainting hyetometers, while energy is usually calculated by regression models, which were obtained through typical measurements among rainfall energy, rainfall intensity and rainfall quantity.

Obviously, obtain rainfall physical parameters through stain method, calculate rainfall energy through a regression mode is unscientific when adopted in large region, because this method tends to neglect spatial and temporal variation of rain, will unavoidably reduce the measurement accuracy. Moreover, stain method's accuracy and efficiency are limited because it depends a lot on human measurement. As a result, there is still an urgent demand to develope an instrument which can measure rainfall physical parameters timely, thus creating a method to calculate rainfall energy rapidly in soil erosion subject.

## The principle of rainfall energy calculation formula derivation

### Theoretical basis for rainfall energy calculation

From physics, rainfall is the process of raindrops movement with their potential energy turn to energy. Raindrops potential energy come from geopotential energy earth gravitation, with the landing of drops, their potential energy turn to be energy. If a rain drop is regarded as a particle, individual rain drop's energy can be calculated according to physical basic laws if its quantity and velocity are known.

Where E is individual rain drop's energy (J), m is its quality (kg) and v the velocity (m/s).

### Derivation for rainfall energy calculation formula

Every single rain drop's measurement is limited by special uniformity because special variability of rainfall is inevitable. In order to avoid measurement errors caused by rainfall special variability, this paper introduces the concept of rainfall measure field, which is a special region, raindrops in this region can be recognized as uniformitied. Meanwhile, water's density is 1 kg/m^3^, and raindrops' density is also considered to be 1 kg/m^3^ when the change of raindrops' density is neglected. Then, according to the above description from formula *(5)*, in a special rainfall measure field, an individual rain drop's energy can be calculated as follows:

Where e_1_ is an individual rain drop's energy (J/ an individual rain drop), m the quantity, d the density (kg/m^3^), b the bulk (m^3^) and v the final velocity of a drop (m/s).

This above description shows that an individual rain drop's energy can be calculated if a drop's volume and final velocity are known, in fact, every measurement are conducted through sampling under a special rainfall measure field, this is to say, raindrops' quantity of every sampling region are determined and countable, this sampling region is called view field of measurement. The total energy (e_2_) of view field of measurement is the sum of all individual raindrops energy.

Where e_2_ is the total energy of view field of measurement (J/m^2^), b is the volume of the drops (m^3^), v the final velocity (m/s) and n the quantities of raindrops.

To an individual rainfall, suppose that the term could be divided into n stages, the first stage's rainfall energy is e_21._ t_1_, the second is e_22._ t_2_, ..., the n-th is e_2n._ t_n_, then, the total energy of a individual view field of measurement (e_3_) is the sum of all stages' energy.

Where e_3_ is the total energy of a individual view field of measurement (J/m^2^), e_21_, e_22_, e_23_, ..., e_2n_ is the rainfall energy (J/mm) from the first drop to the n-th drop, t_1_, t_2_, t_3_,..., t_n_ (s) is the interval time of adjacent two rainfall stages.

In order to resolve errors introduced by rainfall special variability, several measurement points must be layouted, the density of measurement points depend on rainfall fields' distribution. That is to say, within the measurement field, measurement points are need as long as this place's rainfall characters are different. An united area's united rainfall precipitation's energy (e_4_) can be calculated by the product of above measurement points' average and convert coefficient (K), which turn the view field of measurement's area to be united area, is expressed as follows:

Where e_4_ is the energy of a unit area's rainfall (J/m^2^), K is the convert coefficient, p are the Places of points, and e_3_ (J/m^2^) is same as above.

Above are about formulas to calculate the energy of a unit area, the results show that, in the given boundary condition, a unit area's energy can be calculated through science calculation as long as raindrops' bulk (b) and final velocity (v) are known. As a result, how to measure these two physical parameters is the key to calculate rainfall energy.

## Measurement of raindrops' physical parameters

### Choice Digital Single Lens Reflex (DSLR) with high definition and high shutter speed

Obtain raindrops images as they fall is the key to measure their bulk (b) and final velocity. Through market research and screening, we know that the exposure velocity of DSLR has reached to 1/10000s~1/300s, it is satisfied to take raindrops' images. Experiments have proved that if an object's diameter is 3 mm with speed of 2 m/s, use DSLR with 1/10000s' exposure velocity to take its static images is appropriate, the error of rain's volume can be controlled under ±7%, when use the DSLR with 1/300s exposure velocity to take rain's tailing images, raindrops' velocity error can be controlled under ±17%. Thus, use DSLR to take raindrops' images as they fall, combine with computer images analysis properties and rainfall process automatic control, raindrops physical parameters can be obtained accurately.

### Manufacture raindrops sampler

**Figure 1:**
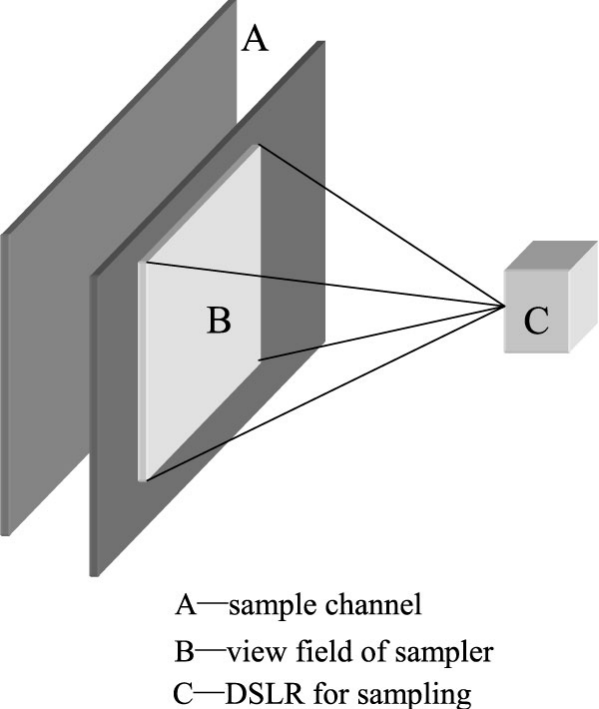
**Structural diagram of raindrops sampler**.

Raindrops sampler is used to collect drops, which are placed in rainfall field, 0.5~1.0m higher than ground when sampling so that raindrops can through sampler continuously and naturally under the precondition of nature rainfall process is not be disturbed. Structural diagram of raindrops sampler is as follows:

The core of raindrops sampler design is sample channel and view field of sampler's geometry size (Figure 1). Generally, the raindrops sampler should be designed under these principles: not only the rainfall distribution of sampling channel can represent the rainfall's average situation, but also there are no overlapping rainfall projections in the view field of sampler's profile. Many experiments have proved that the appropriate size of view field of sampler is 30.0 cm × 30.0 cm, and that the size of sample is 1.0 cm × 5.0 cm. Due to the raindrops density variation in different rainfall intensity, width variable design is need to adjust and calibrate timely according to rainfall intensity in practical measurements in order to satisfy the requirement that there are no overlapping rainfall projection in the profile of view field of sampler.

### Software development of raindrop images analysis and calculation

Two kinds of images of view field of sampler in the same time can be obtained by different DSLR, one of them are taken by DSLR with 1/10000s exposure velocity, raindrops in these images can be considered as static, raindrops distribution and every drops' geometrical size can be obtained by analysing of these images. The other images are taken by DSLR with 1/300s exposure velocity, drops in these images have tail, final velocity of raindrops' can be calculated according to the length of tail and its development time.

**Figure 2:**
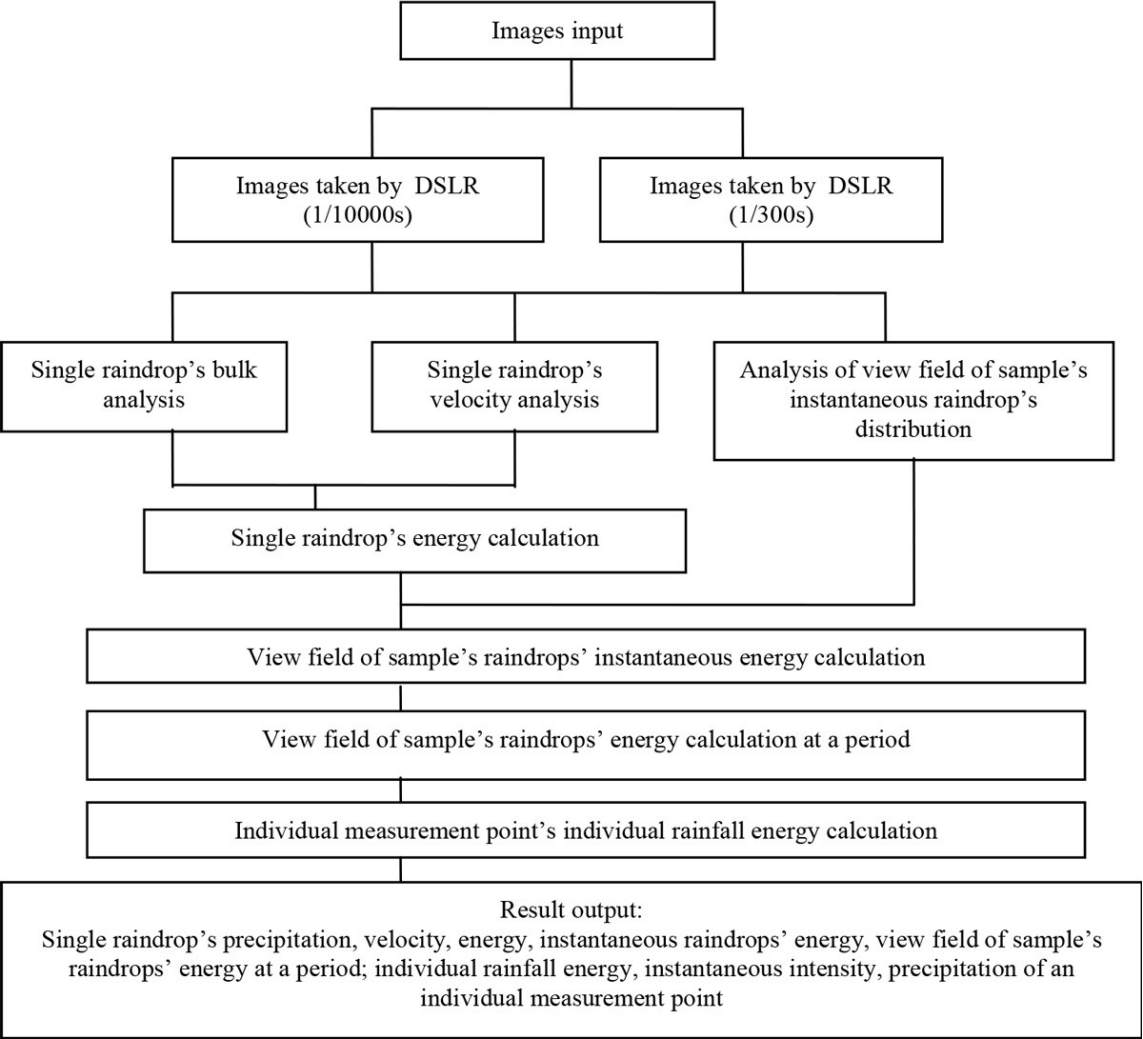
**Flow diagram for raindrop images analysis and calculation software**.

Raindrops physical parameters such as bulk, velocity, rainfall energy, rainfall intensity and rainfall precipitation et al. can be calculated according to view field of sampler's image parameters and corresponding environment parameters. Figure 2 provides a flow diagram for raindrop images analysis and calculation software.

## Conclusions and discussions

Raindrops' physical parameters measurement is a cosmopolitan problem and still has not been resolved. Based on automatic theory control, photogrammetric technique, image analytic, science calculation theory and technology, this paper provides a new idea to measure the physical parameters of raindrops, and then to calculate rainfall characters such as erosivity factor (R), rainfall velocity and so on, which could offer a method of data acquisition and calculation for soil erosion process mechanism study, supply an experimental technique and method to study how rainfall factors act on soil erosion more accurate. However, it is necessary to explain that this research is just in the stage of conceptual model, more deeply researches must be taken in many steps. In order to provide useful information for the interesting researchers, there provide summaries as follows:

1. It is very hard to develop a sampler which can represent raindrops real physical parameters because of the variability of natural rainfall "pattern". How to adjust sample channel width in different rain intensity so that there are no overlapping rainfall projection in the view field of sampler's profile will directly affect accuracy of raindrops physical parameters measurement and calculation. To resolve this problem, not only a lot of experiments are required to determine appropriate sample channel width in different rain styles, but also the adjustability is demanded in the progress of sampler integration.
2. The above descriptions are about measurement progression of a measuring point, However, a certain region's individual rainfall physical parameters and characters need multi-point repeat measurements to determine measure points and its distribution in the uneven rainfall fields.
3. To a region rain measurement, its rainfall physical parameters and characters can be measured through successive measurements through above method, however, this involve a tremendous amount of work. So, how to establish relationships between typical measurements and previous data such as rainfall intensity, precipitation and so on, then calculating rainfall physical parameters and characters according to these established relationships is a great issue need deepen study in future.
